# Risk factors for fatal outcome in patients with opioid dependence treated with methadone in a family medicine setting in Croatia

**DOI:** 10.3325/cmj.2013.54.42

**Published:** 2013-02

**Authors:** Venija Cerovečki, Hrvoje Tiljak, Zlata Ožvačić Adžić, Miljenko Križmarić, Peter Pregelj, Andrej Kastelic

**Affiliations:** 1 Department for Family Medicine, University of Zagreb, School of Medicine, Zagreb, Croatia; 2 University of Maribor, Faculty of Medicine, Maribor, Slovenia; 3 University Psychiatric Hospital Ljubljana, Ljubljana Polje, Slovenia; 4 University of Ljubljana, Faculty of Medicine, Ljubljana, Slovenia; 5 University Psychiatric Hospital Ljubljana, Center for Treatment of Drug Addiction, Ljubljana, Slovenia

## Abstract

**Aim:**

To determine the risk factors for fatal outcome in patients with opioid dependence treated with methadone at the primary care level.

**Methods:**

A group of 287 patients with opioid dependence was monitored prospectively from 1995 to 2007. At the beginning of the study, we collected the data on patient baseline characteristics, treatment characteristics, and living environment. At the annual check-up, we collected the data on daily methadone dose, method of methadone therapy administration, and family physician’s assessment of the patient’s drug use status.

**Results:**

Out of 287 patients, 8% died. Logistic regression analysis showed that the predictors of fatal outcome were continuation of drug use during previous therapeutic attempts (odds ratio [OR], 19.402; 95% confidence interval [CI], 1.659-226.873), maintenance therapy as the planned treatment modality (OR, 3.738; 95% CI, 1.045-13.370), living in an unstable relationship (OR, 9.275; 95% CI, 2.207-38.984), and loss of continuity of care (OR, 12.643; 95% CI, 3.001-53.253).

**Conclusion:**

The patients presenting these risk factors require special attention. It is important for family physicians to insist on compliance with the treatment protocol and intervene when they lose contact with the patient to prevent the fatal outcome.

Opioid dependence is a chronic and relapsing disorder with a high risk of health problems arising from direct drug effects, injecting-related injuries, and dependence-associated lifestyle factors (1,2). Patients with opioid dependence have a 6-20 times higher death rate than their non-dependent peers (3). The major causes of mortality are opioid-related overdose, transmission of blood-borne viruses (HIV, HCV), as well as some environmental and contextual factors (4). Heroin, a still widely available and predominant illicit opioid, can elicit respiratory depression and coma (5). The most widely known pharmacologic treatment for opioid dependence, that with methadone, retains patients in treatment and decreases illegal drug use (6). However, it is also known that methadone treatment is associated with potentially fatal side effects (7).

Treatment of patients with opioid dependence in Croatia is organized as a shared care model including family physicians at the primary care level, psychiatrists in specialized drug treatment centers at the community level, and hospital psychiatrists at the secondary health care level (8). Family physicians provide easy access to treatment, continuity of care, and a comprehensive and holistic approach, while psychiatrists provide specific knowledge and skills necessary for the treatment of opioid dependence. The treatment of patients with opioid dependence in the family medicine setting in Croatia is the same as for any other chronic disease; it offers insight into the overall health status, treatment monitoring, and a good follow up of treatment outcomes (9). This approach increases patient safety by individual health risk management, daily contacts with the patients, consultations with the families, and monitoring of all prescriptions issued to the patients (10).

Despite a number of short-term studies, little is known about the long-term effects of this approach and factors associated with fatal outcome. Therefore, the aim of the present study was to determine the risk factors for fatal outcome in patients with opioid dependence treated with methadone in the family medicine setting in Croatia.

## Material and methods

The study prospectively followed-up patients with opioid dependence treated with methadone in the family medicine settings in Novi Zagreb from January 31, 1995 to January 31, 2007. In that time, methadone was the only opiate agonist registered for treatment of opioid dependence in Croatia. A total of 287 patients, 232 male and 55 female, with opioid dependence were monitored by 56 family physicians. Data were collected through personal interviews with family physicians, as well as by examining personal medical information sheets and the accompanying medical records.

At the beginning of the study, data were collected on the patients’ basic personal characteristics, treatment characteristics, and patients’ living environment.

The basic characteristics included sex, age, age at the beginning of drug/heroin use, age of first treatment, methadone dosage at the beginning of treatment, and duration of drug/heroin abuse before therapy. Treatment characteristics included therapeutic attempts before the initiation of the methadone-based treatment followed in this study (therapy-naïve, treated with methadone without any remissions, treated with methadone until remission, other medications) and the treatment modality (detoxification or maintenance therapy). The data on the patient’s living environment included information on whether the patient lived alone, in a stable relationship, or in an unstable relationship.

The data collected at the annual check-up included the daily methadone dose, the method of methadone therapy administration (self-administration, administration by someone else, or combined administration), and the family physician’s assessment of the patient’s drug use status.

In the case of drug use status, the family physician inquired whether the patient attained abstinence without methadone treatment, whether the patient attained abstinence under methadone treatment, whether the patient continued to use drugs despite methadone treatment, or whether the family physician lost contact with the patient and could not assess the patient’s drug use status. The study was approved by the Ethics Committee of the University of Zagreb School of Medicine (No 04 - 77/2010 - 24).

### Statistics

Statistical analysis was performed to test for the differences between the patients with fatal outcome and patients without fatal outcome. Shapiro-Wilk test was used to check for normality of distribution of the continuous variables. We performed descriptive analysis, bivariate analysis (Man Whitney U test for numerical variables and exact test with Monte Carlo approximation, due to unbalanced data for categorical variables) and multivariate analysis (logistic regression), which included all the variables that were significant in bivariate analysis.

In logistic regression analysis, fatal outcome was used as the dependent variable, with a number of other predictor variables. Predictor variables included treatment characteristics-previous therapeutic attempts, treatment characteristics-planned treatment modality, patient’s living environment, and family physician’s assessment of the patient’s drug use status. *P* < 0.05 was considered significant. Data were analyzed using statistical software IBM SPSS, version 20 (SPSS Inc., Chicago, IL, USA).

## Results

Twenty three patients died, only one of whom was female, and 54 women and 210 men survived. No significant differences in sex were observed between the groups (*P* = 0.092, exact test). The 12-year mortality rate was 8% and average annual mortality rate was 0.7%. For patients who died, the median (range) length of follow-up before death was 7 years (1-12, inter-quartile range [IQR], 4-9 years). There were no significant differences between the groups in drug consumption profile at the beginning of methadone treatment (baseline characteristics) (Table 1).

**Table 1:** Drug consumption profile at the beginning of methadone treatment for surviving and deceased patients (baseline characteristics)

Baseline characteristics of patients at the beginning of methadone treatment	Living (n = 264)		Dead (n = 23)		P*
	Median	Range	Median	Range	
Age of first drug use (years)	17	12-33	18	12-23	0.312
Age of first heroin use (years)	20	15-35	20	16-27	0.499
Age when methadone treatment was first started (years)	26	17-45	26	17-37	0.709
Daily methadone dose administered at the beginning of methadone treatment (mg)	50	0-200	40	15-100	0.952
Length of involvement in a drug addict community prior to methadone treatment (years)	8	0-27	8	2-19	0.640
Length of involvement in a heroin addict community prior to methadone treatment (years)	6	0-26	5	1-17	0.594

*Man Whitney U test.

There were differences in previous therapeutic attempts (Table 2). Significantly more surviving patients were therapy-naïve before the treatment initiation or had undergone a previous methadone therapy, which resulted in a remission. Significantly more patients who died had undergone methadone therapy without success (*P* = 0.001, Table 2).

**Table 2:** Treatment characteristics for surviving and deceased patients

Treatment characteristics	Living		Dead	
	No.	%	No.	%
Previous therapeutic attempts:*				
therapy-naïve	164	62.1	10	43.5
methadone treatment without any remission	34	12.9	11	47.9
methadone treatment until remission	53	20.1	1	4.3
other medications	13	4.9	1	4.3
**Planned treatment modality:^†^**				
detoxification	239	90.5	15	65.2
maintenance therapy	25	9.5	8	34.8

*P = 0.001, exact test.

†*P* = 0.002, exact test.

The prevailing planned treatment modality for both groups was detoxification (Table 2). However, maintenance therapy was planned for significantly more patients with fatal outcome (N = 8, 34.8%) than for surviving patients (N = 25, 9.5%) (*P* = 0.002, Table 2).

Significant differences were observed in the patient’s living environment (*P* = 0.002, exact test). Out of 264 survivors, 57 (21.6%) lived alone, 194 (73.5%) lived in a stable relationship, and 13 (4.9%) lived in an unstable relationship. Out of 23 patients with fatal outcome, 8 (34.8%) lived alone, 10 (43.5%) lived in a stable relationship, and 5 (21.7%) lived in an unstable relationship.

There was no significant difference between the median daily dose of methadone administered to the surviving patients in the last study year and that administered to the patients with fatal outcome in the year before their death (50 mg, 0-200 mg, for each patient group) (Mann-Whitney U-test, *P* = 0.872).

There was also no significant difference in the method of methadone therapy administration in the last study year for surviving patients and in the year before the death for the deceased patients (*P* = 0.623, exact test). Among 264 surviving patients, 44 (16.7%) self-administered methadone, 26 (9.8%) were administered methadone by someone else, and 194 (73.5%) used a combined model. Out of 23 patients who died, 5 (21.7%) self-administered methadone, 1 (4.3%) was administered methadone by someone else, and 17 (74%) used a combined model.

Significant differences were observed in family physician’s assessment of the patient’s drug use status in the last study year for survivors and in the year preceding death for patients who died (Table 3). Significantly more patients who died continued to abuse drugs despite treatment (34.8% vs 19.3%) and lost contact with the family physician (30.4% vs 8.3%), while significantly more surviving patients attained abstinence from drug use under treatment (58.8% vs 17.4%) (*P* < 0.001, Table 3).

**Table 3:** Family physician’s assessment of the drug use status for surviving and deceased patients

Family physician’s assessment of the patient's drug use status*	Living^†^		Dead^‡^	
	No.	%	No.	%
Abstinence attained without methadone treatment	36	13.6	4	17.4
Abstinence attained under methadone treatment	155	58.8	4	17.4
Abusing drugs despite methadone treatment	51	19.3	8	34.8
Assessment lacking due to loss of contact with the family physician	22	8.3	7	30.4

*P < 0.001, exact test.

†Family physician’s assessment of drug use status in the last year of the study.

‡Family physician’s assessment of drug use status in the year before the patent’s death.

Multivariate logistic regression analysis showed that fatal outcome was predicted by methadone treatment without any remission during previous therapeutic attempts (odds ratio [OR], 19.402; 95% confidence interval [CI], 1.659-226.873), maintenance therapy as the planned treatment modality (OR, 3.738; 95% CI, 1.045-13.370), living in an unstable relationship (OR, 9.275; 95% CI, 2.207-38.984), and lack of the family physician's assessment of the patient’s drug use status due to loss of contact (OR, 12.643; 95% CI, 3.001-53.253) (Table 4).

**Table 4:** Logistic regression analysis on the association of fatal outcome as dependent variable and treatment characteristics, patient's living environment and family physician's assessment of the patient's drug use status as independent variables

Independent variables	Odds Ratio	95% confidence interval	*P*
**Treatment characteristics – previous therapeutic attempts:**			
methadone treatment until remission	1.000		
methadone treatment without any remission	19.402	1.659-226.873	0.018
therapy-naïve	7.505	0.746-75.487	0.087
other medications	18.045	0.744-437.886	0.075
**Treatment characteristics – planned treatment modality:**			
detoxification	1.000		
maintenance therapy	3.738	1.045-13.370	0.043
**Patient's living environment:**			
living in a stable relationship	1.000		
living alone	1.529	0.501-4.668	0.456
living in an unstable relationship	9.275	2.207-38.984	0.002
**Family physician’s assessment of the patient's drug use status:**			
abstinence attained under methadone treatment	1.000		
abstinence attained without methadone treatment	3.037	0.652-14.152	0.157
abusing drugs despite methadone treatment	3.491	0.886-13.749	0.074
assessment lacking due to lost contact with the family physician	12.643	3.001 -53.253	0.001
constant	0.006		<0.001

*χ^2^_9_ = 43 020, overall model *P* < 0.001, Nagelkerke R^2^ = 0.325.

## Discussion

The 12-year mortality rate in the present study was 8% and average annual mortality rate was 0.7%. Mortality rate was lower than in other studies (3,5,11). The 12-month mortality rate in a long-term study in primary care and specialized substitution centers in Germany was 1.1% (11).

A failure to achieve remission despite previous methadone treatment at the start of the study represented the strongest predictor of fatal outcome in this study. Additionally, the plan to undergo methadone maintenance treatment at the start of the study was also a predictor of the fatal outcome. This indicates that patients at risk for fatal outcome were the patients with more severe opioid dependence, as previously reported (12,13). It could be expected that the severity of the opioid dependence would also be reflected by the daily methadone dose (14). However, there was no significant difference between the groups in the daily methadone dose administered either at the beginning or at the end of treatment. These findings could be partially explained by the Croatian treatment model: implementation of treatment primarily in non-hospital settings characterized by a high degree of accessibility to care, which had to be balanced with relatively lower methadone doses in order to assure safety of the treatment in the out-patient setting (8). To support this thesis, the endpoint methadone dose in this study was 50 mg for both groups of patients, which was lower than the daily methadone dose required for stable maintenance, which ranges from 60 mg to 100 mg, as recommended in the literature (15,16).

The practical implication of our research is that the family physician who is initiating methadone substitution therapy for a new opioid dependent patient should analyze the results of the previous therapeutic attempts, since the failure of previous methadone therapy can be considered as a risk factor for a fatal outcome. In the Croatian shared care model, the decision on the planned treatment modality (detoxification or maintenance therapy) is made by the psychiatrist, with the family physician having a less active role. Nonetheless, patients treated with maintenance therapy should also be considered by the family physician as patients under higher risk for fatal outcome. Family medicine is a suitable context for patient-centered approach, ie, recognizing individual risk factors in opioid dependent patients in care (17). We might conclude that patients in whom maintenance therapy is planned should be more intensively monitored by their family physicians, as would patients with any other severe chronic disease.

The lack of family physician’s assessment of the patient’s drug use status due to loss of contact represented the second strongest predictor of fatal outcome. This finding reveals the importance of family physicians’ insisting on compliance and adequate intervention in case of contact loss with the patient. The family physician has to continuously assess the drug use status of methadone-treated patients and, if they fail to attain abstinence or their management is impossible, consider their referral to the psychiatrist in order to revise the therapeutic plan. It is important to point out that during the study period family physicians lost contact with only 8.3% of the surviving patients and 30.4% of the deceased ones. In similar surveys from other countries, the continuity of care was significantly worse. Since retention in the treatment program is a key indicator of methadone treatment success (18,19), this fact indicates a high quality of care in the family medicine setting in Croatia. A study in Ireland reported that 61% of patients included in methadone treatment remained in continuous treatment for more than 1 year, and those who were treated by a specialist were twice as likely to leave methadone treatment in 1 year than those who were treated by a primary care physician (18).

Previous studies suggested that the key features of a successful method of retaining opioid users in treatment are an adequate methadone dose and easy access to the primary care physician (18). Our study supports the approach of treating patients with opioid dependence in the same way as patients with any other chronic disease (20). Family physicians are particularly important for such a treatment since continuity of care is central to family physicians’ everyday work, especially in treating chronic patients from vulnerable and socially deprived groups (21,22).

Living in an unstable relationship represented the third strongest predictor of fatal outcome in this study. However, it is not clear whether this observation is connected with severity of the illness at the time of first evaluation or whether living in an unstable relationship increases the risk of other, unrecognized medical conditions that might increase the possibility of fatal outcome. The significant relationship between family background and opioid dependence development is well known (23). A recent 12-year follow-up study of patients in methadone treatment observed that moderate and long-term recovery was associated with fewer relationship disruptions (24). Our findings should therefore serve as an incentive to family physicians to monitor and motivate harmonious family functioning.

There was no significant difference between the groups in drug consumption profile at the beginning of methadone treatment. This finding might be explained by a sudden huge rise in drug supply and demand in Croatia in the mid-nineties (8). The male to female ratio in the study was 4.2 to 1, which is in line with the sex ratio in national and international observational studies (25,26), suggesting that our group was representative of the studied population. Our patients abused heroin for 5 to 6 years before the beginning of therapy, while the European Monitoring Centre for Drugs and Drug Addiction Annual Report for the year 2008 found this period to be on average 8 years (26). The relatively early beginning of treatment of patients observed in this study could have contributed to better results for continuity of care. The time between the onset of drug abuse and start of treatment represents the time frame in which family physicians play an indispensable role in the early detection of opioid dependence, as well as in motivating patients with opioid dependence to undergo treatment.

There was no significant difference between the groups in the method of methadone therapy administration in the last study year for surviving patients and in the year before death for deceased patients. These findings suggest that adherence to the standardized procedures for methadone therapy administration, implemented both by patients and their family physicians, improved patients’ safety (15,27).

One of the study limitations is the sample size. The total number of participants in the deceased group was too small to examine the association between the mortality risk and other factors. Second, there are several methodological concerns. As in similar studies, the use of proxy informants, retrospective data collection, lack of blinding regarding case and comparison participants, and the potential impact of opioid dependence and the stigma of opioid dependence on reporting may have influenced the reliability of the data. Also, our strategy of scheduling interviews with patients could have increased the risk of recall bias. The data should be interpreted having these limitations in mind.

In conclusion, the observed risk factors should be taken into consideration by family physicians in their everyday work with opioid-dependent patients treated with methadone.
